# P-693. RSV Pneumonia Incidence in Solid Organ Transplant Recipients from 2014 to 2023 in an US Collaborative Network

**DOI:** 10.1093/ofid/ofae631.889

**Published:** 2025-01-29

**Authors:** German Contreras, Georgy Golovko

**Affiliations:** University of Texas Medical Branch, Houston, Texas; University of Texas Medical Branch, Houston, Texas

## Abstract

**Background:**

Few studies have assessed the burden of RSV disease in solid organ transplant recipients (SOTr). The aim of our study is to determine the cumulative incidence (CI) and outcomes of RSV pneumonia among SOTr over 10 years.

Cumulative Incidence of RSV Pneumonia in SOTr 2014-2023

**Figure:**
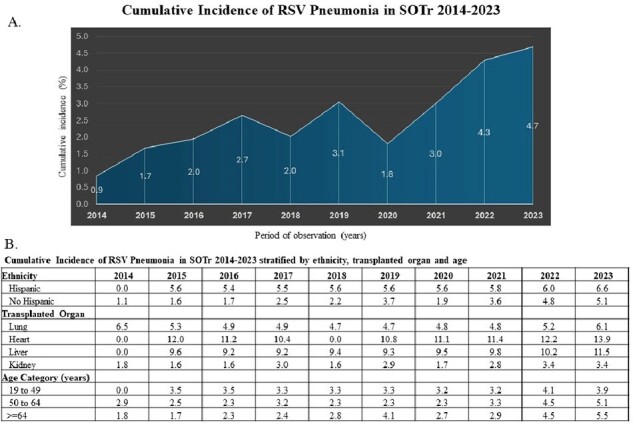

**Methods:**

We used the TriNetX platform to access aggregated and de-identified EHRs from healthcare organizations in the USA from January 1^st^ of 2014 to December 31^st^ of 2023. We included individuals ≥18 years old with history of Heart, Lung, Kidney, or Liver transplantation. RSV infection status was based on RSV laboratory testing and clinical diagnosis codes (CPT and ICD-10, respectively). The index event was defined as the day of the first positive RSV test. The primary outcomes of interest were ICU admission, Invasive Mechanical ventilation, and all-cause mortality 30-days after index event. We used the TriNetX built-in functions to perform analysis.

**Results:**

A total of 34,301 SOTr recipients were tested for RSV and 1671 were positive for RSV infection.

The population was mainly Male (53%) and No Hispanic (87%) with a median age at the time of diagnosis of 56 years. A high proportion of the cases were diagnosed in the South (37%) and Midwest (24%).

The CI of RSV Pneumonia significantly increased from 2014 to 2023, and it was higher among Hispanic, individuals ≥ 64 years old and among heart transplant recipients (Figure A-B).

During the observation period 137 (8.2%) were admitted to ICU, 99 (5.9%) required mechanical ventilation and 455 (27.2%) had complications with the transplanted organ during the RSV episode and 42 (2.5%) died.

**Conclusion:**

We found evidence of substantial ethnic and age inequalities in the incidence of RSV pneumonia in SOTr recipients.

**Disclosures:**

**All Authors**: No reported disclosures

